# Is consanguinity a risk factor for developing Alzheimer's disease and related dementia? A feasibility study in an under‐represented sample from Pakistan

**DOI:** 10.1002/alz70856_102452

**Published:** 2025-12-24

**Authors:** Maheen Mausoof Adamson, Muhammad Adeel Parvaz, Saqib Bakhshi, Salman Kirmani, Shireen Najam, Momin Kazi, Paul M. Thompson

**Affiliations:** ^1^ WOMEN CoE, VA Palo Alto/Neurosurgery, Stanford, Palo Alto, CA, USA; ^2^ Mount Sinai, New York, NY, USA; ^3^ Aga Khan University Hospital, Karachi, Sindh, Pakistan; ^4^ University of Southern California, Los Angeles, CA, USA

## Abstract

**Background:**

Pakistan is the fifth most populous country, with an estimated 150,000–200,000 patients with Alzheimer's disease and related dementia (ADRD). Additionally, more than 60% of all unions in Pakistan are consanguineous, potentially posing a greater impact on genetically transmitted diseases like ADRD. Consanguinity, although prevalent in densely populated low‐ and middle‐income countries, is gravely underrepresented in current international AD research initiatives. The scientific premise of this project is to conduct the first international clinical research collaboration to study the association between consanguinity and AD.

**Method:**

The current feasibility study, in collaboration with Aga Khan University Hospital (Karachi, Pakistan) and the NIA‐funded Alzheimer's Disease Sequencing Project (ADSP), will implement genetic and phenotypic research for ADRD in Pakistan that is consistent with ADSP goals and standards for early detection of ADRD. First, the ENIGMA‐PAK team will be trained by ADSP investigators on collecting blood, phenotypic and neuroimaging data that is consistent with ADSP and ADNI protocols and data standards. Data will be collected from 120 participants diagnosed with ADRD and 80 age‐ and sex‐matched controls (50% female; age range 60‐80 years) from urban and peri‐urban sites located in the province of Sindh. Demographic, genotype and phenotype data will be shared with ADSP cores for comparison with other international samples. We will also provide MRI data from 10 individuals to ENIGMA for additional comparisons.

**Result:**

In this presentation, we will share feasibility data from this unique sample to increase diversity in ADRD research and to further our understanding of the role of consanguinity in ADRD.

**Conclusion:**

Including a Pakistani cohort, provides a unique opportunity to further diversify genomic studies of ADRD and enhances the combined power of neuroimaging and non‐neuroimaging biomarkers to study the prevalence and progression of AD in various countries.

